# 2-Benzoyl-1-(2,4-dichloro­phen­yl)-3-phenyl­guanidine

**DOI:** 10.1107/S160053680903387X

**Published:** 2009-08-29

**Authors:** Ghulam Murtaza, Masahiro Ebihara, Muhammad Said, M. Khawar Rauf, Saeed Anwar

**Affiliations:** a Department of Chemistry, Quaid-i-Azam University, Islamabad 45320, Pakistan; b Department of Chemistry, Faculty of Engineering, Gifu University, 1-1 Yanagido, Gifu City 501-1193, Japan; c Department of Chemistry, Abdul Wali Khan University, Mardan, Pakistan

## Abstract

In the title compound, C_20_H_15_Cl_2_N_3_O, a typical polysubstituted guanidine with normal geometric parameters, the torsion angles [C—N—C—O = 3.8 (2), N—C—N—C = −6.1 (2)°] indicate that the guanidine and carbonyl groups are almost coplanar, due to the pseudo-hexa­gonal ring formed by intra­molecular N—H⋯O hydrogen bonds. The crystal packing is stabilized by inter­molecular N—H⋯O hydrogen bonds, which link the mol­ecules into centrosymmetric dimers.

## Related literature

The guanidinium group is present in diverse biologically active substances, see: Manimala & Anslyn (2002 ▶); Berlinck (2002 ▶). These compounds have received increasing inter­est as medicinal agents, for example having an effect on the neuromuscular junction, see: Rodrigues-Simioni *et al.* (1997 ▶). Guanidine derivatives are also useful building blocks in synthetic organic chemistry, see: Costa *et al.* (1998 ▶); Kovacevic & Maksic (2001 ▶), and due to their strongly basic character, guanidines can be considered as super-bases for biological systems, see: Ishikawa & Isobe (2002 ▶). For related structures, see: Cunha *et al.* (2005 ▶); Murtaza *et al.* (2007 ▶, 2008 ▶, 2009 ▶). For the preparation of *N*-benzoyl-*N*′-phenyl­thio­urea, see: Rauf *et al.* (2009 ▶).
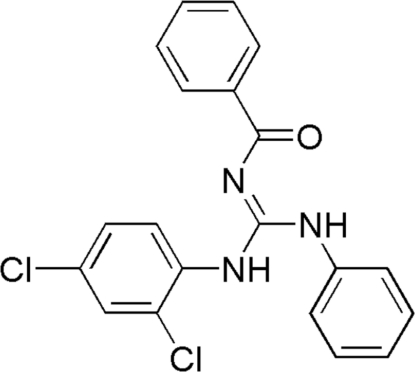


## Experimental

### 

#### Crystal data

C_20_H_15_Cl_2_N_3_O
*M* *_r_* = 384.25
Monoclinic, 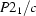

*a* = 16.461 (6) Å
*b* = 6.663 (2) Å
*c* = 19.388 (6) Å
β = 124.072 (5)°
*V* = 1761.0 (10) Å^3^
*Z* = 4
Mo *K*α radiation
μ = 0.38 mm^−1^
*T* = 123 K
0.42 × 0.40 × 0.18 mm

#### Data collection

Rigaku/MSC Mercury CCD diffractometer
Absorption correction: none
13586 measured reflections
4023 independent reflections
3768 reflections with *I* > 2σ(*I*)
*R*_int_ = 0.031

#### Refinement

*R*[*F* ^2^ > 2σ(*F* ^2^)] = 0.042
*wR*(*F* ^2^) = 0.099
*S* = 1.12
4023 reflections
241 parameters
H atoms treated by a mixture of independent and constrained refinement
Δρ_max_ = 0.34 e Å^−3^
Δρ_min_ = −0.27 e Å^−3^

### 

Data collection: *CrystalClear* (Molecular Structure Corporation & Rigaku, 2001 ▶); cell refinement: *CrystalClear*; data reduction: *TEXSAN* (Molecular Structure Corporation & Rigaku, 2004 ▶); program(s) used to solve structure: *SIR97* (Altomare *et al.*, 1999 ▶); program(s) used to refine structure: *SHELXL97* (Sheldrick, 2008 ▶); molecular graphics: *ORTEPII* (Johnson, 1976 ▶) and *ORTEP-3* (Farrugia, 1997 ▶); software used to prepare material for publication: *SHELXL97* and *TEXSAN*.

## Supplementary Material

Crystal structure: contains datablocks I, global. DOI: 10.1107/S160053680903387X/hg2555sup1.cif

Structure factors: contains datablocks I. DOI: 10.1107/S160053680903387X/hg2555Isup2.hkl

Additional supplementary materials:  crystallographic information; 3D view; checkCIF report

## Figures and Tables

**Table 1:** Hydrogen-bond geometry (Å, °)

*D*—H⋯*A*	*D*—H	H⋯*A*	*D*⋯*A*	*D*—H⋯*A*
N3—H3⋯O1	0.84 (2)	2.01 (2)	2.6471 (19)	132.7 (18)
N3—H3⋯O1^i^	0.84 (2)	2.36 (2)	3.032 (2)	138.2 (18)

Symmetry code: (i) 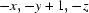.

**Fig. 1.**
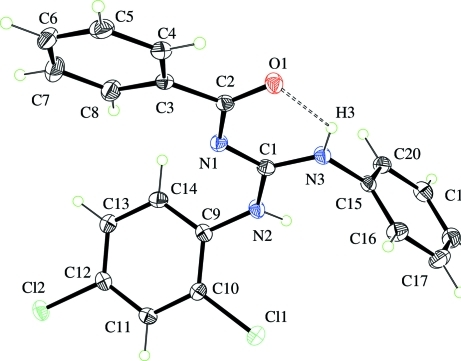
Molecular structure of (I) showing atom numbering scheme. Displacement ellipsoids are drawn at the 50% probability level.

**Fig. 2.**
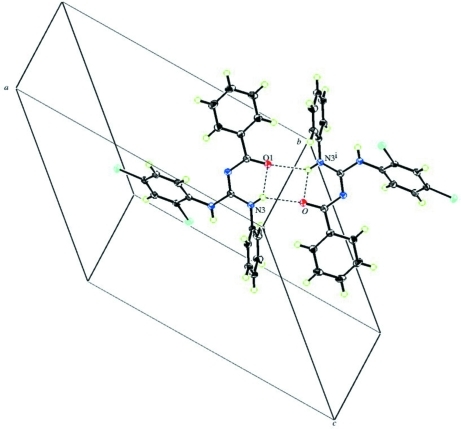
Hydrogen-bonded dimer structure of (I). Hydrogen bonds shown as dashed lines.

## supplementary crystallographic information

### Comment

Polysubstituted guanidines is a field of intense investigation as guanidinium group is present in diverse biologically active substances (Manimala & Anslyn, 2002; Berlinck, 2002) These compounds have received increasing interest as medicinal agents, e.g it has effect on the neuromuscular junction (Rodrigues-Simioni *et al.*, 1997). In addition to their biological role, guanidine derivatives are very useful building blocks in synthetic organic chemistry (Costa *et al.*, 1998; Kovacevic & Maksic, 2001). Due to their strongly basic character, guanidines can be considered as super-bases for the biological systems (Ishikawa & Isobe, 2002). The title compound (I), (Fig.1) is a typical *N,N',N"*-tri-substituted guanidine with normal geometric parameters (Cunha *et al.*, 2005; Murtaza *et al.*, 2007, 2008, 2009). The C3—O1 bond shows expected full double bond character while the short values for C1—N1, C2—N1, C2—N2 and C2—N3 bonds indicate partial double bond character. The dihedral angles between the guanidine plane [C2/N1/N2/N3] and the mean planes of phenyl rings C3–C8, C9–C14 & C15–C20 are 22.23 (11)°, 48.06 (7)° & 83.53 (7)°, respectively. The guanidine moiety and carbonyl group are almost co-planar as reflected by the torsion angles [C1—N1—C2—O1 = 3.8 (2)° and N3—C1—N1—C2= -6.1 (2)°], due to the presence of intramolecular N—H···O hydrogen bonding (Table 1), forming a six-membered ring commonly observed in this class of compounds (Cunha *et al.*, 2005). The crystal packing shows intermolecular N—H···O hydrogen bonds which link the molecules into centrosymmetric dimers (Fig. 2).

### Experimental

*N*-Benzoyl-*N*'-phenylthiourea (0.256 g, 1 mmol) was prepared (Rauf *et al.*, 2009) and dissolved in 10 ml of dimethylformamide and taken into two neck round bottom flask. 2,4-dichloroaniline (0.16 g, 1 mmol) and triethylamine (0.28 ml, 2 mmol) were added and the mixture was stirred well below 5°C. Mercuric chloride (0.272 g, 1 mmol) was then added and mixture was vigorously stirred for 15 h till the completion of reaction as monitored by TLC. When all the thiourea was consumed, 20 ml of chloroform was added and the suspension was filtered through sintered glass funnel to remove residual HgS formed as a byproduct during the reaction. The solvent was evaporated under reduced pressure and residue was dissolved in 20 ml of CH_2_Cl_2_. Other byproducts were extracted out with water (4×30 ml). The organic phase was dried over anhydrous MgSO_4_ and then filtered. The solvent was evaporated and product was further purified by column chromatography. The target guanidine was recrystallized in ethanol to obtain single crystals suitable for X-ray analysis.

### Refinement

Positional parameters of the H atoms bonded to N were refined with *U*_iso_(H) = 1.2U_eq_(N). Hydrogen atoms bonded to C were included in calculated positions and refined as riding on their parent C atom with C—H = 0.95 Å and *U*_iso_(H) = 1.2U_eq_(C).

### Crystal data

**Table:**

C_20_H_15_Cl_2_N_3_O	*F*(000) = 792
*M**_r_* = 384.25	*D*_x_ = 1.449 Mg m^−^^3^
Monoclinic, *P*2_1_/*c*	Mo *K*α radiation, λ = 0.71070 Å
Hall symbol: -P 2ybc	Cell parameters from 5226 reflections
*a* = 16.461 (6) Å	θ = 3.1–27.5°
*b* = 6.663 (2) Å	µ = 0.38 mm^−^^1^
*c* = 19.388 (6) Å	*T* = 123 K
β = 124.072 (5)°	Block, colourless
*V* = 1761.0 (10) Å^3^	0.42 × 0.40 × 0.18 mm
*Z* = 4	

### Data collection

**Table:**

Rigaku/MSC Mercury CCD diffractometer	3768 reflections with *I* > 2σ(*I*)
Radiation source: fine-focus sealed tube	*R*_int_ = 0.031
graphite	θ_max_ = 27.5°, θ_min_ = 3.3°
Detector resolution: 14.62 pixels mm^-1^	*h* = −21→16
ω scans	*k* = −8→8
13586 measured reflections	*l* = −17→25
4023 independent reflections	

### Refinement

**Table:**

Refinement on *F*^2^	Primary atom site location: structure-invariant direct methods
Least-squares matrix: full	Secondary atom site location: difference Fourier map
*R*[*F*^2^ > 2σ(*F*^2^)] = 0.042	Hydrogen site location: inferred from neighbouring sites
*wR*(*F*^2^) = 0.099	H atoms treated by a mixture of independent and constrained refinement
*S* = 1.12	*w* = 1/[σ^2^(*F*_o_^2^) + (0.0364*P*)^2^ + 1.087*P*] where *P* = (*F*_o_^2^ + 2*F*_c_^2^)/3
4023 reflections	(Δ/σ)_max_ < 0.001
241 parameters	Δρ_max_ = 0.34 e Å^−^^3^
0 restraints	Δρ_min_ = −0.27 e Å^−^^3^

### Special details

**Table:**

Geometry. All esds (except the esd in the dihedral angle between two l.s. planes) are estimated using the full covariance matrix. The cell esds are taken into account individually in the estimation of esds in distances, angles and torsion angles; correlations between esds in cell parameters are only used when they are defined by crystal symmetry. An approximate (isotropic) treatment of cell esds is used for estimating esds involving l.s. planes.
Refinement. Refinement of F^2^ against ALL reflections. The weighted R-factor wR and goodness of fit S are based on F^2^, conventional R-factors R are based on F, with F set to zero for negative F^2^. The threshold expression of F^2^ > 2sigma(F^2^) is used only for calculating R-factors(gt) etc. and is not relevant to the choice of reflections for refinement. R-factors based on F^2^ are statistically about twice as large as those based on F, and R- factors based on ALL data will be even larger.

### Fractional atomic coordinates and isotropic or equivalent isotropic displacement parameters (Å^2^)

**Table:**

	*x*	*y*	*z*	*U*_iso_*/*U*_eq_	
C1	0.16683 (11)	0.5401 (2)	0.00837 (10)	0.0159 (3)	
N1	0.23055 (9)	0.3930 (2)	0.05737 (8)	0.0168 (3)	
N2	0.27838 (10)	0.1383 (2)	0.15357 (9)	0.0190 (3)	
H2	0.2660 (14)	0.070 (3)	0.1846 (13)	0.023*	
N3	0.12213 (10)	0.2596 (2)	0.09071 (9)	0.0176 (3)	
H3	0.0802 (15)	0.344 (3)	0.0586 (13)	0.021*	
C2	0.20779 (11)	0.2689 (2)	0.09780 (10)	0.0159 (3)	
O1	0.08319 (8)	0.57114 (18)	−0.00799 (7)	0.0198 (2)	
C3	0.20711 (11)	0.6783 (2)	−0.02684 (10)	0.0169 (3)	
C4	0.16230 (12)	0.8635 (3)	−0.05922 (10)	0.0197 (3)	
H4	0.1064	0.9001	−0.0594	0.024*	
C5	0.19875 (13)	0.9949 (3)	−0.09131 (11)	0.0244 (4)	
H5	0.1687	1.1222	−0.1121	0.029*	
C6	0.27903 (13)	0.9406 (3)	−0.09309 (11)	0.0270 (4)	
H6	0.3033	1.0292	−0.1159	0.032*	
C7	0.32341 (14)	0.7558 (3)	−0.06128 (12)	0.0294 (4)	
H7	0.3782	0.7180	−0.0626	0.035*	
C8	0.28878 (13)	0.6261 (3)	−0.02765 (11)	0.0233 (4)	
H8	0.3206	0.5011	−0.0050	0.028*	
C9	0.37728 (11)	0.1401 (2)	0.17925 (10)	0.0160 (3)	
C10	0.45195 (12)	0.1401 (2)	0.26411 (10)	0.0162 (3)	
C11	0.55020 (11)	0.1405 (2)	0.29205 (10)	0.0170 (3)	
H11	0.6005	0.1393	0.3499	0.020*	
C12	0.57271 (11)	0.1428 (2)	0.23292 (11)	0.0173 (3)	
C13	0.50041 (12)	0.1377 (2)	0.14848 (10)	0.0186 (3)	
H13	0.5175	0.1366	0.1091	0.022*	
C14	0.40293 (12)	0.1341 (2)	0.12201 (10)	0.0184 (3)	
H14	0.3530	0.1276	0.0642	0.022*	
Cl1	0.42229 (3)	0.14079 (6)	0.33735 (2)	0.02082 (11)	
Cl2	0.69542 (3)	0.15526 (6)	0.26631 (3)	0.02167 (11)	
C15	0.10396 (11)	0.1209 (2)	0.13708 (10)	0.0160 (3)	
C16	0.11539 (12)	0.1817 (3)	0.21063 (11)	0.0216 (3)	
H16	0.1332	0.3162	0.2295	0.026*	
C17	0.10044 (13)	0.0438 (3)	0.25644 (11)	0.0259 (4)	
H17	0.1086	0.0843	0.3069	0.031*	
C18	0.07378 (12)	−0.1519 (3)	0.22886 (11)	0.0237 (4)	
H18	0.0639	−0.2454	0.2605	0.028*	
C19	0.06150 (12)	−0.2117 (3)	0.15488 (11)	0.0224 (4)	
H19	0.0423	−0.3454	0.1355	0.027*	
C20	0.07740 (11)	−0.0753 (3)	0.10929 (10)	0.0191 (3)	
H20	0.0701	−0.1165	0.0592	0.023*	

### Atomic displacement parameters (Å^2^)

**Table:**

	*U*^11^	*U*^22^	*U*^33^	*U*^12^	*U*^13^	*U*^23^
C1	0.0155 (7)	0.0168 (7)	0.0129 (7)	−0.0014 (6)	0.0065 (6)	−0.0022 (6)
N1	0.0163 (6)	0.0181 (7)	0.0157 (6)	0.0015 (5)	0.0086 (5)	0.0019 (5)
N2	0.0148 (6)	0.0207 (7)	0.0200 (7)	0.0019 (5)	0.0089 (6)	0.0060 (6)
N3	0.0143 (6)	0.0184 (7)	0.0177 (7)	0.0020 (5)	0.0074 (5)	0.0049 (6)
C2	0.0155 (7)	0.0152 (7)	0.0140 (7)	0.0002 (6)	0.0064 (6)	−0.0012 (6)
O1	0.0161 (5)	0.0210 (6)	0.0226 (6)	0.0033 (4)	0.0110 (5)	0.0056 (5)
C3	0.0158 (7)	0.0205 (8)	0.0114 (7)	−0.0024 (6)	0.0058 (6)	−0.0008 (6)
C4	0.0186 (7)	0.0226 (8)	0.0143 (7)	0.0000 (6)	0.0070 (6)	0.0013 (6)
C5	0.0273 (8)	0.0230 (9)	0.0166 (8)	−0.0022 (7)	0.0085 (7)	0.0035 (7)
C6	0.0302 (9)	0.0326 (10)	0.0199 (8)	−0.0075 (8)	0.0152 (7)	0.0026 (8)
C7	0.0284 (9)	0.0380 (11)	0.0301 (10)	0.0001 (8)	0.0215 (8)	0.0038 (8)
C8	0.0233 (8)	0.0271 (9)	0.0223 (8)	0.0037 (7)	0.0145 (7)	0.0039 (7)
C9	0.0147 (7)	0.0123 (7)	0.0183 (8)	0.0014 (6)	0.0076 (6)	0.0019 (6)
C10	0.0195 (7)	0.0139 (7)	0.0170 (7)	0.0002 (6)	0.0113 (6)	0.0008 (6)
C11	0.0160 (7)	0.0146 (7)	0.0159 (7)	0.0009 (6)	0.0062 (6)	0.0007 (6)
C12	0.0149 (7)	0.0139 (7)	0.0212 (8)	0.0011 (6)	0.0089 (6)	0.0005 (6)
C13	0.0204 (8)	0.0170 (8)	0.0199 (8)	0.0025 (6)	0.0123 (7)	0.0023 (6)
C14	0.0186 (7)	0.0174 (8)	0.0151 (7)	0.0010 (6)	0.0069 (6)	0.0007 (6)
Cl1	0.0237 (2)	0.0222 (2)	0.0199 (2)	0.00056 (15)	0.01430 (17)	0.00129 (15)
Cl2	0.01518 (19)	0.0240 (2)	0.0251 (2)	0.00091 (14)	0.01083 (16)	0.00198 (16)
C15	0.0118 (6)	0.0189 (8)	0.0151 (7)	0.0017 (6)	0.0062 (6)	0.0035 (6)
C16	0.0253 (8)	0.0186 (8)	0.0193 (8)	−0.0010 (6)	0.0114 (7)	−0.0014 (7)
C17	0.0311 (9)	0.0315 (10)	0.0180 (8)	0.0018 (8)	0.0155 (7)	0.0019 (7)
C18	0.0219 (8)	0.0255 (9)	0.0235 (9)	0.0023 (7)	0.0126 (7)	0.0087 (7)
C19	0.0199 (8)	0.0178 (8)	0.0260 (9)	−0.0009 (6)	0.0106 (7)	0.0015 (7)
C20	0.0173 (7)	0.0218 (8)	0.0162 (8)	0.0006 (6)	0.0082 (6)	0.0001 (6)

### Geometric parameters (Å, °)

**Table:**

C1—O1	1.2447 (19)	C9—C10	1.397 (2)
C1—N1	1.359 (2)	C10—C11	1.388 (2)
C1—C3	1.504 (2)	C10—Cl1	1.7412 (17)
N1—C2	1.329 (2)	C11—C12	1.388 (2)
N2—C2	1.367 (2)	C11—H11	0.9500
N2—C9	1.411 (2)	C12—C13	1.384 (2)
N2—H2	0.86 (2)	C12—Cl2	1.7439 (17)
N3—C2	1.339 (2)	C13—C14	1.384 (2)
N3—C15	1.433 (2)	C13—H13	0.9500
N3—H3	0.84 (2)	C14—H14	0.9500
C3—C4	1.393 (2)	C15—C20	1.388 (2)
C3—C8	1.397 (2)	C15—C16	1.390 (2)
C4—C5	1.390 (2)	C16—C17	1.392 (3)
C4—H4	0.9500	C16—H16	0.9500
C5—C6	1.389 (3)	C17—C18	1.384 (3)
C5—H5	0.9500	C17—H17	0.9500
C6—C7	1.388 (3)	C18—C19	1.390 (3)
C6—H6	0.9500	C18—H18	0.9500
C7—C8	1.383 (3)	C19—C20	1.391 (2)
C7—H7	0.9500	C19—H19	0.9500
C8—H8	0.9500	C20—H20	0.9500
C9—C14	1.392 (2)		
			
O1—C1—N1	127.45 (15)	C11—C10—C9	121.57 (15)
O1—C1—C3	119.26 (14)	C11—C10—Cl1	118.64 (13)
N1—C1—C3	113.27 (13)	C9—C10—Cl1	119.79 (13)
C2—N1—C1	119.92 (13)	C10—C11—C12	117.98 (15)
C2—N2—C9	125.14 (14)	C10—C11—H11	121.0
C2—N2—H2	117.2 (13)	C12—C11—H11	121.0
C9—N2—H2	115.8 (13)	C13—C12—C11	121.75 (15)
C2—N3—C15	122.90 (14)	C13—C12—Cl2	119.34 (13)
C2—N3—H3	115.4 (14)	C11—C12—Cl2	118.91 (12)
C15—N3—H3	121.7 (14)	C12—C13—C14	119.28 (16)
N1—C2—N3	126.61 (14)	C12—C13—H13	120.4
N1—C2—N2	117.82 (14)	C14—C13—H13	120.4
N3—C2—N2	115.56 (14)	C13—C14—C9	120.69 (15)
C4—C3—C8	119.04 (15)	C13—C14—H14	119.7
C4—C3—C1	119.33 (14)	C9—C14—H14	119.7
C8—C3—C1	121.63 (15)	C20—C15—C16	120.33 (15)
C5—C4—C3	120.48 (16)	C20—C15—N3	119.68 (15)
C5—C4—H4	119.8	C16—C15—N3	119.97 (15)
C3—C4—H4	119.8	C15—C16—C17	119.43 (16)
C6—C5—C4	120.14 (17)	C15—C16—H16	120.3
C6—C5—H5	119.9	C17—C16—H16	120.3
C4—C5—H5	119.9	C18—C17—C16	120.42 (17)
C7—C6—C5	119.44 (17)	C18—C17—H17	119.8
C7—C6—H6	120.3	C16—C17—H17	119.8
C5—C6—H6	120.3	C17—C18—C19	119.99 (16)
C8—C7—C6	120.71 (17)	C17—C18—H18	120.0
C8—C7—H7	119.6	C19—C18—H18	120.0
C6—C7—H7	119.6	C18—C19—C20	119.90 (16)
C7—C8—C3	120.18 (17)	C18—C19—H19	120.1
C7—C8—H8	119.9	C20—C19—H19	120.1
C3—C8—H8	119.9	C15—C20—C19	119.93 (16)
C14—C9—C10	118.64 (15)	C15—C20—H20	120.0
C14—C9—N2	121.60 (14)	C19—C20—H20	120.0
C10—C9—N2	119.71 (15)		
			
O1—C1—N1—C2	−3.8 (3)	N2—C9—C10—C11	179.58 (14)
C3—C1—N1—C2	174.94 (14)	C14—C9—C10—Cl1	−178.31 (12)
C1—N1—C2—N3	6.2 (2)	N2—C9—C10—Cl1	−0.7 (2)
C1—N1—C2—N2	−172.70 (14)	C9—C10—C11—C12	0.6 (2)
C15—N3—C2—N1	−179.82 (15)	Cl1—C10—C11—C12	−179.13 (12)
C15—N3—C2—N2	−0.9 (2)	C10—C11—C12—C13	−2.3 (2)
C9—N2—C2—N1	8.0 (2)	C10—C11—C12—Cl2	177.02 (12)
C9—N2—C2—N3	−171.02 (15)	C11—C12—C13—C14	1.3 (2)
O1—C1—C3—C4	15.7 (2)	Cl2—C12—C13—C14	−177.99 (12)
N1—C1—C3—C4	−163.16 (14)	C12—C13—C14—C9	1.4 (2)
O1—C1—C3—C8	−164.54 (16)	C10—C9—C14—C13	−3.0 (2)
N1—C1—C3—C8	16.6 (2)	N2—C9—C14—C13	179.45 (15)
C8—C3—C4—C5	−0.5 (2)	C2—N3—C15—C20	−82.0 (2)
C1—C3—C4—C5	179.28 (15)	C2—N3—C15—C16	96.34 (19)
C3—C4—C5—C6	1.5 (3)	C20—C15—C16—C17	0.3 (2)
C4—C5—C6—C7	−1.1 (3)	N3—C15—C16—C17	−178.04 (15)
C5—C6—C7—C8	−0.3 (3)	C15—C16—C17—C18	−0.4 (3)
C6—C7—C8—C3	1.3 (3)	C16—C17—C18—C19	−0.2 (3)
C4—C3—C8—C7	−0.9 (3)	C17—C18—C19—C20	0.9 (3)
C1—C3—C8—C7	179.35 (16)	C16—C15—C20—C19	0.4 (2)
C2—N2—C9—C14	−53.8 (2)	N3—C15—C20—C19	178.77 (14)
C2—N2—C9—C10	128.62 (17)	C18—C19—C20—C15	−1.0 (2)
C14—C9—C10—C11	2.0 (2)		

### Hydrogen-bond geometry (Å, °)

**Table:**

*D*—H···*A*	*D*—H	H···*A*	*D*···*A*	*D*—H···*A*
N3—H3···O1	0.84 (2)	2.01 (2)	2.6471 (19)	132.7 (18)
N3—H3···O1^i^	0.84 (2)	2.36 (2)	3.032 (2)	138.2 (18)

Symmetry codes: (i) −*x*, −*y*+1, −*z*.
